# CAPIRI-IMRT: a phase II study of concurrent capecitabine and irinotecan with intensity-modulated radiation therapy for the treatment of recurrent rectal cancer

**DOI:** 10.1186/s13014-015-0360-5

**Published:** 2015-02-28

**Authors:** Gang Cai, Ji Zhu, Joshua D Palmer, Ye Xu, Weigang Hu, Weilie Gu, Sanjun Cai, Zhen Zhang

**Affiliations:** Department of Radiation Oncology, Shanghai Cancer Center, Shanghai Medical College, Fudan University, 270 Dong An Road, Shanghai, China; Department of Radiation Oncology, Kimmel Cancer Center, Jefferson Medical College, Thomas Jefferson University, Philadelphia, PA USA

**Keywords:** Recurrent rectal cancer, Irinotecan, Capecitabine, Intensity-modulated radiation therapy

## Abstract

**Background:**

This study investigated the local effect and acute toxicity of irinotecan and capecitabine with concurrent intensity-modulated radiation therapy (IMRT) for the treatment of recurrent rectal cancer without prior pelvic irradiation.

**Methods:**

Seventy-one patients diagnosed with recurrent rectal cancer who did not previously receive pelvic irradiation were treated in our hospital from October 2009 to July 2012. Radiotherapy was delivered to the pelvis, and IMRT of 45 Gy (1.8 Gy per fraction), followed by a boost of 10 Gy to 16 Gy (2 Gy per fraction), was delivered to the recurrent sites. The concurrent chemotherapy regimen was 50 mg/m^2^ irinotecan weekly and 625 mg/m^2^ capecitabine twice daily (Mon-Fri). Radical surgery was recommended for medically fit patients without extra-pelvic metastases. The patients were followed up every 3 months. Tumor response was evaluated using CT/MRIs according to the RECIST criteria or postoperative pathological findings. NCI-CTC 3.0 was used to score the toxicities.

**Results:**

Forty-eight patients (67.6%) had confirmed recurrent rectal cancer without extra pelvic metastases, and 23 patients (32.4%) had extra pelvic metastases. Fourteen patients (19.7%) underwent radical resections (R0) post-chemoradiation. A pathologic complete response was observed in 7 of 14 patients. A clinical complete response was observed in 4 patients (5.6%), and a partial response was observed in 22 patients (31.0%). Only 5 patients (7.0%) showed progressive disease during or shortly after treatment. Of 53 symptomatic patients, clinical complete and partial symptom relief with chemoradiation was achieved in 56.6% and 32.1% of patients, respectively. Only 2 patients (2.8%) experienced grade 4 leukopenia. The most common grade 3 toxicity was diarrhea (16 [22.5%] patients). The median follow-up was 31 months. The cumulative local progression-free survival rate was 74.2% and 33.9% at 1 and 3 years after chemoradiation, respectively. The cumulative total survival rate was 80.1% and 36.5% at 1 and 3 years after chemoradiation, respectively.

**Conclusions:**

This study revealed that concurrent irinotecan and capecitabine with IMRT significantly relieves local symptoms and exhibits promising efficacy with manageable toxicities in recurrent rectal cancer without prior pelvic irradiation. Improving the rate of R0 resections will be investigated in a future study.

## Introduction

Recent advances in pretreatment radiologic evaluation, radiation and chemotherapy, and surgical techniques, such as total mesorectal excision (TME), have led to a declining incidence of locoregional recurrences for patients with primary rectal cancer [1,2]. Nevertheless, some patients still develop pelvic recurrence with or without extra pelvic metastases, especially in cases that did not receive standard initial treatment. Generally, pelvic recurrences portend a poor prognosis and are morbid, leading to troublesome symptoms, including pelvic pain, bleeding, and bowel obstruction [3,4].

Currently, there is no consensus for the treatment of recurrent rectal cancer. Although radical surgery remains the only therapy with curative potential, only approximately 20-30% of patients with recurrent rectal cancer can undergo R0 resection [5]. Most patients cannot undergo curative surgery due to locally unresectable disease, medical unfitness, an unwillingness to accept the considerable associated morbidity and mortality or additional unresectable pelvic metastases. There is no survival benefit of an R2 resection compared with no resection [6].

Several studies have shown that radiotherapy (RT) is an effective method of treatment for recurrent rectal cancer, and a positive relationship between radiation dose and clinical outcome in recurrent rectal cancer has also been observed [7,8]. Therefore, there is a need to adopt new techniques, such as intensity-modified radiotherapy (IMRT), stereotactic body RT, or proton and heavy ion RT, to safely deliver higher doses of radiation and improve local control. The combination of RT and chemotherapy with curative intent or a (neo) adjuvant setting improves the clinical outcome of patients with recurrent rectal cancer [9]. However, the optimal regimens for concurrent chemoradiation have not been clearly defined. Radiation sensitization with capecitabine has been shown to be effective for the treatment of rectal cancer. FOLFOX is currently the standard adjuvant chemotherapy; thus, most recurrent patients have previously received oxaliplatin. Both preclinical and clinical studies have revealed a synergistic effect of CPT-11 or irinotecan when combined with radiation and remarkable radiosensitizing activity of CPT-11 [10]. Several small studies have evaluated CPT-11 and capecitabine combined with radiation and demonstrated that this treatment is well tolerated and effective for primary rectal cancer [11-15]. However, no previous prospective study has evaluated the efficacy and safety of irinotecan and capecitabine concurrent with IMRT for treatment of recurrent rectal cancer. Based on these considerations, we carried out a phase II trial to investigate the local effect and acute toxicity of irinotecan and capecitabine concurrent with IMRT for the treatment of recurrent rectal cancer.

## Materials and methods

### Patients

Seventy-one patients with recurrent rectal cancer were enrolled between October 2009 and November 2011. This prospective study was approved by our institutional review board (Fudan University Shanghai Cancer Center), and all patients provided informed consent.

Eligibility criteria included patients with histologically confirmed primary rectal adenocarcinoma; age between 18 and 75 years; Eastern Cooperative Oncology Group (ECOG) performance score ≤2; adequate hematological, liver function, and other laboratory parameters (leucocytes >4.0 × 10^9^/L, platelets >100 × 10^9^/L, bilirubin <1.5 the upper limit of normal range (ULN); aspartate aminotransferase/alanine aminotransferase ≤2.5 × ULN; and serum creatinine <1.25 × ULN).

All patients were discussed among our multidisciplinary team (MDT) before treatment and diagnosed with a pelvic recurrence either by histological confirmation or typical appearance on PET/CT, computed tomography (CT) or MRI imaging. Measurable lesions were observed in the field of RT.

Exclusion criteria were as follows: prior pelvic irradiation; prior chemotherapy with irinotecan in the last six months; any other malignancy; significant coronary or cardiac conditions; serious uncontrolled infection(s); a psychiatric disorder; and pregnancy or lack of contraception use in women with child-bearing potential.

### Pretreatment evaluation

The pretreatment workup was conducted within two weeks of the treatment start date and included complete history and physical examination, digital rectal examination, colonoscopy (if possible), tumor biopsy (if possible), chest CT, abdominal CT and pelvic PET/CT or CT or MRI, and complete laboratory tests.

### Radiotherapy

RT was delivered with a linear accelerator using 6-MV photons. Every patient had a planning CT scan in the treatment position. IMRT planning was used for all patients based on the planning CT imaging. The target definition followed the recommendations of ICRU Report No. 83 [16]. The target volumes and nearby organs at risk were delineated on the Pinnacle 8.0 m planning system. The gross tumor volume (GTV) was determined by a combination of the findings on physical exam, CT, MRI, and/or PET-CT. The clinical target volume (CTV) included the GTV, internal iliac, pre-sacral and peri-rectal nodal regions, external iliac nodal region (if recurrent lesions extended into gynecologic/genitourinary structures or positive external iliac lymph nodes) and inguinal nodal region (if recurrent lesions extended to the anal verge, peri-anal skin or positive inguinal nodes). CTV was generated according to the RTOG anorectal contouring atlas, when available [17]. The PTV1 was generated with a 0.5-1 cm asymmetrical margin around the CTV. A 5 mm expansion was used in areas where the CTV was close to the small bowel, bladder and femoral heads, and a 10 mm margin was used elsewhere. The PTV2 was generated by symmetrically expanding the GTV by 2 cm. The small bowel, bladder and femoral heads were defined as organs at risk. Inverse planning with five to seven equally spaced, coplanar IMRT fields were constructed. RT was delivered at a dose of 55–61 Gy in 30–33 treatment fractions, 45 Gy in 25 fractions to PTV1 and 10–16 Gy in 5–8 fractions to PTV2.

### Chemotherapy

Irinotecan and capecitabine were delivered concurrently with RT. Starting at day 1 of RT for the duration of the irradiation (Mon-Fri), patients received capecitabine 625 mg/m^2^ orally within 30 minutes of finishing a meal, as well as weekly irinotecan at 50 mg/m^2^ for five consecutive weeks (days 1, 8, 15, 22, and 29).

### Maintenance chemotherapy and surgery

After concurrent chemoradiotherapy, maintenance chemotherapy was recommended for all patients but was not included in the study protocol. Maintenance chemotherapy for the patients was individualized, with no specific recommendations.

After concurrent chemoradiotherapy, the MDT, which included a group of experienced colorectal surgeons, evaluated the clinical response of the tumor based on imaging and examined the patient to determine whether they were suitable resection candidates.

### Follow-up

All patients with recurrent rectal cancer were evaluated for local responses 3 months after concurrent chemoradiation using CT, MRI, PET/CT or pathologic examination. The patients continued in serial follow up every 3 months with clinical examinations, abdominal and pelvic CTs or MRIs. Tumor response was evaluated using CT/MRIs according to the RECIST criteria or postoperative pathological findings. Adverse events were assessed at least weekly during RT using the National Cancer Institute (NCI) common toxicity criteria (NCI-CTC version 3.0).

### Statistical considerations

The primary efficacy endpoint for this study was local response rate (complete or partial response). Secondary efficacy endpoints included relief of local symptoms, acute toxicity and survival.

The duration of local response and survival were analyzed using SPSS 17.0 statistical software. Survival was analyzed using the Kaplan-Meier method, and comparisons were made using log-rank tests.

## Results

### Patient characteristics

A total of 71 patients with recurrent rectal cancer with a median age of 53 years (range, 30–75 years) were enrolled. Enrolled patients were predominantly male (47 men and 24 women). Isolated pelvic locoregional recurrences occurred in 48 patients (67.6%), whereas 23 patients (32.4%) developed extra pelvic metastases. A total of 101 recurrent sites in all patients were included, 48 cases (67.6%) with single sites of recurrence and 23 cases (32.4%) with multiple recurrences. Thirty-four recurrences (33.7%) were located in the peri-rectal region, 26 (25.7%) in the pre-sacral region, 6 (5.9%) at the external iliac nodal region and 4 (4.0%) at the inguinal nodal region. After concurrent chemoradiation, 56 patients (78.9%) received 5-FU-based maintenance chemotherapy. The patient characteristics are summarized in Table 1.

**Table 1:** **Patient characteristics (n = 71)**

**Characteristics**	**No.**	**%**
Median age (range)	53 years (30–75)	
Gender		
Male	47	66.2
Female	24	33.8
Extra pelvic metastases		
No	48	67.6
Yes	23	32.4
Recurrent sites		
Single	48	67.6
Multiple	23	32.4
Recurrence location (n = 101)		
Peri-rectal region	34	33.7
Pre-sacral region	26	25.7
Internal iliac nodal region	23	22.8
Perineum	8	7.9
External iliac nodal region	6	5.9
Inguinal nodal region	4	4.0

At primary diagnosis, the initial staging was as follows: 6 (8.5%) stage I patients, 13 (18.3%) stage II patients, and 52 (73.2%) stage III patients. A total of 45 patients (63.3%) previously underwent low anterior resection, 25 (35.2%) previously underwent abdominoperineal resection, and 1 (1.4%) previously underwent local excision. In addition, 61 patients (85.9%) previously received postoperative chemotherapy consisting of 12 cycles (range, 2–12) of 5-FU plus oxaliplatin or 6 cycles (range, 6–8) capecitabine plus oxaliplatin.

### Local response and symptom relief

The overall local response rate was 46.5% at 3 months after concurrent chemoradiation. Complete response was observed in 11 patients (15.5%). Fourteen patients (19.7%) underwent radical resections (R0) post-chemoradiation. A pathologic complete response (pCR) was observed in 7 of 14 patients. Complete clinical response was observed in 4 patients (5.6%), and a partial response was observed in 22 patients (31.0%). Stable disease was observed in 33 patients (46.5%). Progressive disease was observed in 5 patients (7%) during or shortly after treatment. Overall, local control was achieved in 93% of patients at 3 months after concurrent chemoradiation (Table 2).

**Table 2:** **Summary of clinical findings**

**Parameter**	**No. of patients**	**%**
Response* (n = 71)		
Pathologic complete response	7	9.9
Clinical complete response	4	5.6
Partial response	22	31.0
Stable disease	33	46.5
Progressive disease	5	7.0
Symptom relief (n = 53)		
Complete relief	30	56.6
Partial relief	17	32.1
No relief	6	11.3

*: 14 patients underwent radical resections (R0) post-chemoradiation.

Of 53 symptomatic patients, clinical complete and partial symptom relief with chemoradiation was achieved in 56.6% and 32.1% of patients, respectively (Table 2).

### Acute toxicity and dose intensity

Table 3 demonstrates the incidence of acute toxicity during concurrent chemoradiation. Only 2 patients experienced grade 4 leukopenia. Diarrhea was the most common grade 3 toxicity. Grade 3 non-hematological toxicity included diarrhea in 22.5%, radiation dermatitis in 9.9% and cystitis in 4.2% of patients. Grade 3 hand-foot skin reactions were rarely observed. Hematological toxicity of grade 3 included leukopenia in 5.6% or patients. No patients had grade 3 thrombocytopenia.

**Table 3:** **Acute toxicity**

**Toxicity**	**Grade 1 No. (%)**	**Grade 2 No. (%)**	**Grade 3 No. (%)**	**Grade 4 No. (%)**
Hematological				
Leukocytopenia	9 (12.7)	6 (8.5)	4 (5.6)	2 (2.8)
Thrombocytopenia	1 (1.4)	0	0	0
Non-hematological				
Diarrhea	21 (29.6)	14 (19.7)	16 (22.5)	0
Cystitis	12 (16.9)	9 (12.7)	3 (4.2)	0
Dermatitis	8 (8.5)	13 (18.3)	7 (9.9)	0
Vomiting	4 (5.6)	3 (4.2)	0	0
Hand-foot syndrome	4 (5.6)	2 (2.8)	0	0

Thirty-seven patients (52.1%) received all planned irinotecan doses (mean relative dose intensity of 75.2%), and 41 patients (57.7%) received all planned capecitabine doses (mean relative dose intensity 82.4%). Sixty-five patients (91.5%) received assigned doses of RT as scheduled.

### Survival

The median follow-up for living patients after completion of chemoradiation was 31 months (range, 6–54 months). At the time of the last follow-up, clinical local control had been achieved in 42 patients (59.2%), and 43 patients developed distant metastases (60.6%). The cumulative local progression-free survival rate was 74.2% (SE, ±5.2%) at 1 year after chemoradiation and 33.9% (SE, ±6.4%) at 3 years after chemoradiation. The median local control time was 25 months. Forty-one patients (57.5%) died, and the cumulative total survival rate was 80.1% (SE, ±4.8%) at 1 year after chemoradiation and 36.5% (SE, ±6.5%) at 3 years after chemoradiation. The median survival time after chemoradiation was 29 months. Of the 14 patients with R0 resections, the cumulative local progression-free survival rate was 92.3% (SE, ±7.4%) at 1 year after chemoradiation and 53.3% (SE, ±15.7%) at 3 years after chemoradiation. Only 5 patients died (35.7%), and the cumulative total survival rate was 100.0% (SE, ±0%) at 1 year after chemoradiation and 59.4% (SE, ±16.0%) at 3 years after chemoradiation.

## Discussion

The medical literature offers little data on the optimum choice of chemotherapeutic agents to administer concurrently with radiation when treating recurrent rectal cancer. The optimal treatment for recurrent rectal cancer has not yet been defined. Patients undergoing R0 resection have the greatest survival advantage following surgery for recurrent rectal cancer [18], but many patients may not be good candidates for further surgery. In addition, approximately 50% of patients with local recurrence have concomitant metastatic disease at the time of diagnosis [19]. Multimodal treatment may offer superior outcomes in the treatment of patients with recurrent rectal cancers [9]. In addition, no prospective trial has evaluated the efficacy and safety of irinotecan and capecitabine concurrent with IMRT for the treatment of recurrent rectal cancer. This study assesses whether IMRT concurrent with irinotecan and capecitabine is effective and safe in patients with recurrent rectal cancer without prior pelvic irradiation. An overall local response rate of 46.5% and a clinical symptom relief rate of 88.7% were achieved. Only 2.8% of patients experienced grade 4 leukopenia. Grade 3 diarrhea, radiation dermatitis, leukopenia and cystitis were observed in 22.5%, 9.9%, 5.6% and 4.2% of patients, respectively.

Some dosimetric and clinical studies have shown that IMRT for rectal cancer can reduce the incidence of irradiated small bowel and treatment-related toxicities compared with standard 3-dimensional conformal RT (3DCRT) [20,21]. Therefore, a relatively higher radiation dose (55–61 Gy) using IMRT was administered to recurrent sites in our study. A number of phase I and II trials have evaluated CPT-11, capecitabine combined with radiation, and demonstrated that this treatment is well-tolerated and effective for primary rectal cancer. From these trials, the recommended irinotecan dose was 40–60 mg/m^2^/week, and the capecitabine dose was 500–825 mg/m^2^, twice daily [11-15]. In our study, concurrent chemotherapy consisted of weekly irinotecan at 50 mg/m^2^ for five consecutive weeks (days 1, 8, 15, 22, and 29), and 625 mg/m^2^ capecitabine was administered orally (weekdays only). The regimen of IMRT concurrent with irinotecan and capecitabine is effective and safe in patients with recurrent rectal cancer.

Our results demonstrated that an overall local control rate of 93% with a local response rate of 46.5% (complete response 15.5%) at 3 months after concurrent chemoradiation and a clinical symptom relief rate of 88.7% were achieved. These findings compare favorably with previous trials. Hu et al. [22] investigated the use of 3DCRT combined with FOLFOX4 chemotherapy in 48 patients with unresectable recurrent rectal cancer. They reported a 47.9% overall response rate in all patients. Only 1 patient achieved a complete response. Another phase II trial [23] evaluated concurrent oxaliplatin and 5-FU with RT in 102 patients with isolated locally recurrent rectal cancer revealing similar results. Complete clinical responses were observed in 13 of the 96 patients (14%). These two trials investigated the addition of oxaliplatin to chemoradiation for the management of locally recurrent rectal cancer. Because most recurrent patients have previously received oxaliplatin after standard adjuvant chemotherapy (FOLFOX), irinotecan as a radiosensitizer in the management of recurrent rectal cancer may be a better treatment choice.

Many phase I and II trials investigating the addition of irinotecan to neoadjuvant chemoradiation for the treatment of locally advanced rectal cancer have been reported, and the pCR rate was ranged from 14% to 26% [12,13,24-26]. In our study, a pathologic complete response was observed in 9.9% (7/71) patients. Our enrolled patients were diagnosed with recurrent rectal cancer along with 32.4% (23/71) patients with distant metastases. The proportion of patients obtaining a clinical symptom relief rate (88.7%) was similar to that reported for some other concurrent chemoradiation protocols (>75%) [27].

Radical resection of recurrent tumors is crucial for long-term cure [5,28,29]. However, only 20% to 30% of all patients with locally recurrent rectal cancer will have a potentially curative operation. In our study, 19.7% (14/71) of all patients and 29.2% (14/48) of the patients without distant metastases underwent R0 resections. Of the 14 patients with R0 resections, only 5 patients died (35.7%), and the cumulative total survival rate was 100.0% (SE, ±0%) at 1 year after chemoradiation and 59.4% (SE, ±16.0%) at 3 years after chemoradiation.

Interestingly, 4 patients with a complete clinical response who did not receive surgery showed good clinical outcomes. Only 1 patient died as a result of distant metastasis, and 3 remained disease free at the time of the analysis. This strategy of close observation for clinical complete responders after preoperative CRT in locally advanced rectal cancer has been discussed [30] but requires further study.

The incidence of acute toxicity with IMRT concurrent with irinotecan and capecitabine for the treatment of recurrent rectal cancer was acceptable and manageable. Diarrhea was the most common acute toxicity. The rate of grade 3 diarrhea (22.5%) in our study was comparable with that reported for irinotecan regimens (11-28%) [12,24,31,32]. Leukocytopenia was the most common hematologic toxicity, with grade 3–4 recorded in 8.5% of patients. This result is similar to that reported previously [10].

The major limitation of the current study was the relatively short median follow-up of 31 months. Additionally, data regarding late toxicity are limited. Moreover, some patients who may have developed late toxicity died prematurely. The long-term efficacy and toxicity may be elucidated upon further follow-up.

In conclusion, concurrent irinotecan and capecitabine with IMRT can significantly relieve local symptoms and exhibits promising efficacy in recurrent rectal cancer without prior pelvic irradiation. Toxicity profiles were acceptable with the dosage and schedule. Improvements in the rate of R0 resections will be investigated in future studies. Neoadjuvant chemoradiation combining newer cytotoxic agents appears promising for the treatment of recurrent rectal cancer.
